# An Ultrasonic Fabrication Method for Epoxy Resin/SbSI Nanowire Composites, and their Application in Nanosensors and Nanogenerators

**DOI:** 10.3390/polym11030479

**Published:** 2019-03-12

**Authors:** Piotr Szperlich, Bartłomiej Toroń

**Affiliations:** Institute of Physics – Center for Science and Education, Silesian University of Technology, Krasinskiego 8 St. 40-019 Katowice, Poland; piotr.szperlich@polsl.pl

**Keywords:** piezoelectric nanocomposite, ultrasound fabrication, nanogenerators, nanosensors, epoxy resin, antimony sulpho-iodide

## Abstract

In this manuscript, a new fabrication technology for epoxy resin/antimony sulpho-iodide (SbSI) nanowire composites is presented. SbSI nanowires, with lateral dimensions of 10 nm to 100 nm and lengths up to several micrometres, have been synthesised using ultrasound irradiation. The prepared SbSI nanowires have been bound with epoxy resin in a mass ratio of 1:4, and then ultrasound irradiation has been used again for homogenization of the mixture. The fabricated epoxy resin/SbSI nanowire composites, due to the piezoelectric properties of SbSI (electromechanical coefficient *k*_33_ = 0.9, and piezoelectric coefficient *d_V_* = 0.9 × 10^−9^ C/N) may be used as an active layer in nanosensors and nanogenerators. The preliminary investigations of epoxy resin/SbSI nanowire composites for sound excitation (frequency *f* = 175 Hz; *L* = 90 dB), vibrations (*f* = 24 Hz; *A* = 1 mm; *F* = 0.73 N), and shock wave (*p* = 6 bar), allowed for the determination of the composite’s open circuit voltage: 0.0153 V_RMS_, 0.166 V_RMS_, and 4.51 V_p-p_, respectively. Maximum power output densities of 0.45 nW/cm^3^ and 860 nW/cm^3^ have been achieved for excitation by sound and vibration, respectively, for a 0.6 mm thick layer of composite.

## 1. Introduction

Here we present a new fabrication technology for an epoxy resin/antimony sulpho-iodide (SbSI) nanowire composite. The first description of the synthesis of antimony sulpho-iodide (SbSI) was given by Henry and Garot in 1824 [1], and in 1958 Mooser and Person predicted the semiconducting properties of A^15^B^16^C^17^ compounds [2]. However, the intensive investigation of SbSI started after a discovery in 1960 by Nitsche and Merz [3] of its photoconductivity, and its piezoelectric and ferroelectric properties were uncovered in 1962 by Fatuzzo et al. [4]. Crystalline SbSI is one of the best piezoelectric materials, with a high-volume piezoelectric modulus of *d_v_* = 0.9 × 10^−9^ C/N [5] and an extremely high electromechanical coupling coefficient of *k*_33_ = 0.90 [6]. Due to these desirable properties, SbSI is a very attractive material for piezoelectric generators and sensors [7,8,9,10].

The applications of SbSI single crystals are limited, due to their reduced mechanical strength. SbSI crystals grow in the shape of needles, due to the presence of double chains [(SbSI)_∞_]_2_, consisting of two chains related by a two-fold screw axis and linked together by short and strong Sb–S bonds [11]. Weak van der Waals-type bonds bind the double chains; these are responsible for the low mechanical strength of the SbSI crystals. However, recently ultrasound irradiation was applied to induce the 1D growth of nanowires in ternary and quaternary chalcohalides, formed from group of 15–16–17 elements [12]. Unfortunately, the application of this technique to SbSI nanowires is limited by the complicated electrical contact preparation to single nanowires. The proposed epoxy resin/SbSI nanowire composites are a solution to the disadvantage mentioned above.

The first piezoelectric nanogenerator for mechanical-to-electrical energy conversion based on zinc oxide nanowires was developed by Wang and Sohn in 2006 [13]. This seminal work created new areas of investigation on such devices—for instance, work on how to harvest acoustic waves [14], vibrations [15,16,17], and other mechanical energy forms [18]. Piezoelectric generators are also used as passive and self-powered sensors [16,19]. Commonly, nanogenerators are prepared as piezoelectric materials deposited or grown across a substrate material [20]. Compression or vibration of the structure, for cases with rigid substrates, or bending of flexible samples leads to voltage generation in the piezoelectric element. Mechanical vibrations are the most common way to induce voltage generation, due to the fact that this form of energy can be easily induced in human environments, i.e., cooking mixers, air conditions, washing machines, refrigerators, and clothes dryers. In order to use these energy sources, it is necessary to construct devices to capture the vibration with frequencies lower than 200 Hz, because such frequencies are typical for everyday machines [21,22]. 

In this manuscript, we present a fabrication method for an epoxy resin/SbSI nanowire composite, and prove that the produced composite shows piezoelectric behaviour that can be used for energy harvesting (using low-frequency vibrations) and sensor technology. 

## 2. Materials and Methods

The SbSI nanowires were prepared from their constituent elements (antimony, sulphur and iodine), weighed in a stoichiometric ratio of 3797 g Sb, 1,000 g S and 3958 g I. Ethanol served as the solvent for this reaction. All used reagents were of analytical purity: antimony (99.95%) purchased from Sigma–Aldrich (Poznan, Poland); sublimated sulphur (pure p.a.); iodine (pure p.a.); and absolute ethanol (pure p.a.), purchased from Avantor Performance Materials, Poland S.A. The elemental mixture was immersed at room temperature and ambient pressure in 20 mL absolute ethanol, which was placed in a polypropylene container, closed by a polyethylene plug in order to avoid outflow of the volatile synthesis products. The cylinder was partly submerged in water in a cup-horn with a 750-Watt ultrasonic VCX-750 processor equipped with a sealed converter VC-334 (Sonics & Materials, Inc., Newtown, OH, USA). The frequency of ultrasound was 20 kHz, and the power density guaranteed by the manufacturer was 565 W/cm^2^. A constant temperature of water (293 K) was kept during the whole process by an AD07R refrigerated circulating bath (PolyScience, Niles, OH, USA).

**Figure 1:**
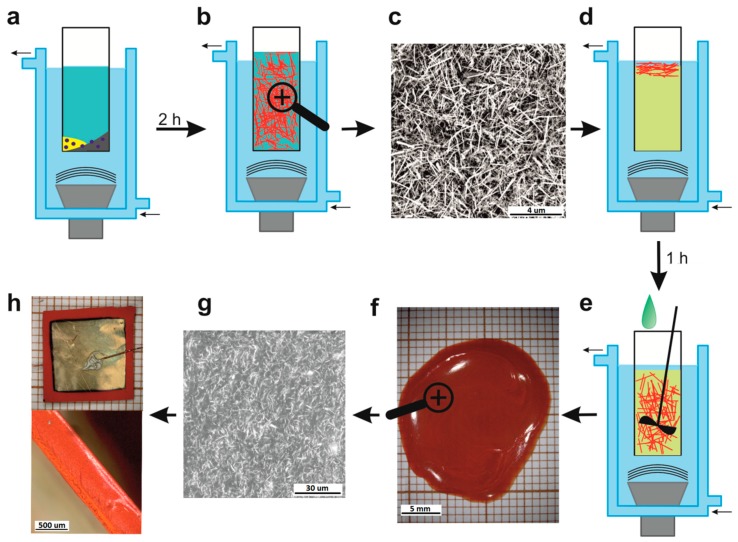
Scheme of sonochemical preparation of the epoxy resin/antimony sulpho-iodide (SbSI) nanowire composite and epoxy resin/SbSI nanowire generator/sensor assembly. The steps are as follows: (**a**) initial elements (sulphur, antimony, and iodine) are sonicated in an ampoule filled with ethanol; (**b**) SbSI nanowires are obtained after 2 h of sonication; (**c**) a typical scanning electron microscopy (SEM) micrograph of the sonochemically prepared SbSI nanowires; (**d**) the epoxy resin and SbSI nanowires (rinsed eight times in ethanol) are sonicated together; (**e**) the addition of hardener to homogenise the mixture of epoxy resin and SbSI nanowires (mechanical and ultrasonic mixing for 5 minutes); (**f**) the obtained epoxy resin/SbSI nanowires composite plate; (**g**) SEM micrograph showing the composite’s morphology; and (**h**) a photo of the piezoelectric epoxy resin/SbSI nanowire generator/sensor (top: the surface with deposited gold electrode and attached electrical leads; bottom: the edge of sample).

During the sonification, a sol was formed. The colour of the slurry changed gradually from red (before sonification) to olive, green, yellow, and then into a red-orange, indicating the growth process of the SbSI nanowires. The process was monitored ex situ by diffusive reflectance spectroscopy (DRS), using a PC-2000 spectrophotometer (Ocean Optics Inc., Winter Park, CO, USA) equipped with an ISP-REF integrating sphere (Ocean Optics Inc., Winter Park, CO, USA). The sonochemical reaction was continued, so as to complete the gelation of the SbSI. When the sonification process was finished, a red-orange gel gave an absorption edge clearly identifiable as SbSI. The whole synthesis process was carried out for a total of 2 h. (Figure 1a)

The obtained gel (Figure 1b) was rinsed with pure ethanol to remove any remaining substrates, and centrifuged to extract the product using an MPW-223e (MPW Med. Instruments, Warsaw, Poland) centrifuge. The process was repeated eight times. Then the SbSI gel was dried under 60 Pa pressure at room temperature for 96 h. The prepared xerogel consisted of SbSI nanowires, with lateral dimensions of 10 nm to 100 nm and lengths up to several micrometres (Figure 1c). 

In the next stage, the SbSI nanowires were added to the LH288 epoxy resin (HAVEL COMPOSITES, Praslavice, Czech Republic) in a mass ratio of 1:4 (5 g SbSI nanowires and 20 g epoxy resin) and mixed (Figure 1d). At first, the mixture was pre-mixed mechanically, but then it was mixed using ultrasound, as previously described, in a cup horn for 60 min. Used resin had an initial viscosity of 0.25 Pa·s. 

After preparing the mixture of SbSI nanowires with epoxy resin, the hardener H281 (HAVEL COMPOSITES, Praslavice, Czech Republic) was added to it. The volume proportion of resin to hardener was 4:1. The whole was mixed mechanically and ultrasonically in previously described set-up configuration (Figure 1e). After the addition of hardener, the working life of the mixture is about 25 min. Within this time, a thin layer of mixture was deposited on a glass substrate and placed in a Bench-top Type Temperature and Humidity Chamber SH-242 (Espec, Osaka, Japan) for 24 h, to be cured at a temperature of 283 K and relative humidity (RH) = 5%. These are good conditions (heat flow, temperature) for a resin curing process (not exceeding 350 K) [23,24]. This is important for the homogeneity and good quality of the produced composite [25,26]. After the main curing step, the composite was removed from substrate and left for 48 hours at room temperature for post-curing. From this method, an epoxy resin/SbSI nanowire composite plate with a surface area of about 3.2 cm^2^, and thickness of 600 μm was obtained (Figure 1f).

The prepared plate was used as an active layer in the fabricated nanogenerators and nanosensors. The 12 x 12 mm^2^ sample was cut from the epoxy resin/SbSI nanowire composite. Thin-film gold electrodes with a surface area of ~0.9 cm^2^ and thickness of 150 nm were deposited on both sides of the sample using a Q150T ES sputter coater (Quorum). The electrodes’ surfaces were slightly smaller than the sample surfaces, to avoid a short circuit between electrodes. Cooper wires were connected to the gold electrodes (Figure 1h) using high-purity silver paste 05002-AB (SPI Supplies). 

The morphology and chemical composition of SbSI nanowires and epoxy resin/SbSI nanowire composites were investigated using a Phenom PRO X scanning electron microscope (Phenom World B.V., Eindhoven, The Netherlands) equipped with an energy-dispersive X-ray spectroscopy (EDS) detector (Phenom World B.V., Eindhoven, The Netherlands).

The temperature characteristics of the sample current were measured in an ambient atmosphere. The sample was mounted on a micro-refrigerator (MMR Technologies, Inc., San Jose, CA, USA), and the temperature was controlled by a K-20 controller (MMR Technologies, Inc., San Jose, CA, USA). The DC current was registered using a Keithley 6517A electrometer (Tektronix, Inc., Beaverton, OR, USA), which was simultaneously used as the voltage source.

The functional parameters (generated voltage, current, and power) of the epoxy resin/SbSI nanowires composite were measured at room temperature in air. A loudspeaker biased with an MXG-9802 function generator (METEX) was used as the acoustic wave source. Samples were located on a testing table while the loudspeaker was placed directly over them, avoiding any influence from housing vibration on the response characteristics. A T-01 sound level meter (Sonopan, Bialystok, Poland) was used to measure the sound pressure level (*L*). The open circuit voltages generated in the epoxy resin/SbSI nanowire composites were recorded using an EG&G 5110 dual phase lock in amplifier (Princeton Applied Research, Oak Ridge, TN, USA). Measurements of the U-I characteristics for different load resistances were performed using a Zeal decade resistance box (1 Ω–1 GΩ).

A plexiglass plate attached to a vibration generator was used as the source of vibration. The sample was mounted on it using a thin layer of wax. The frequency and amplitude of the vibration was measured with a WH-30 vibrometer equipped with an FO-1 octave filter (STANMARK Products, Cracow, Poland). Measurements of the generated voltages were performed, utilizing the same equipment as used in the sound excitation.

The LabView program was developed for computer control of the experiment, data acquisition and analysis.

A CP88 airgun (CarlWalther GmbH, Arnsberg, Germany) equipped with a 12 g CO_2_ capsule was used to check the electric response of the epoxy resin/SbSI nanowire composite to rapid changes in pressure. The pressure of CO_2_ shock waves was measured using a S-10 pressure sensor (WIKA Alexsander Wiegand SE & Co. KG, Klingenberg am Main, Germany). The open-circuit voltage response of the shock pressure was measured using a Photon+ dynamic signal analyser (Bruel & Kjær, Nærum, Denmark).

A permanent magnet shaker LDS V201, with peak sine force ratings of 17.8 N guaranteed by the manufacturer (Bruel & Kjær, Nærum, Denmark) and an LDS LPA100 Linear Power Amplifier (Bruel & Kjær, Nærum, Denmark), was used to measure the response of the epoxy resin/SbSI nanowire composite to impact. The sample was mounted on a plane surface perpendicularly to the moving tip of the shaker. Measurements were performed using a Bench-top Type Temperature and Humidity Chamber SH-242 (Espec, Osaka, Japan) over various temperatures. The open-circuit voltage responses were measured using a Photon+ dynamic signal analyser (Bruel & Kjær, Nærum, Denmark). The temperature range was limited by the working ambient temperature of the shaker. 

## 3. Results 

**Figure 2:**
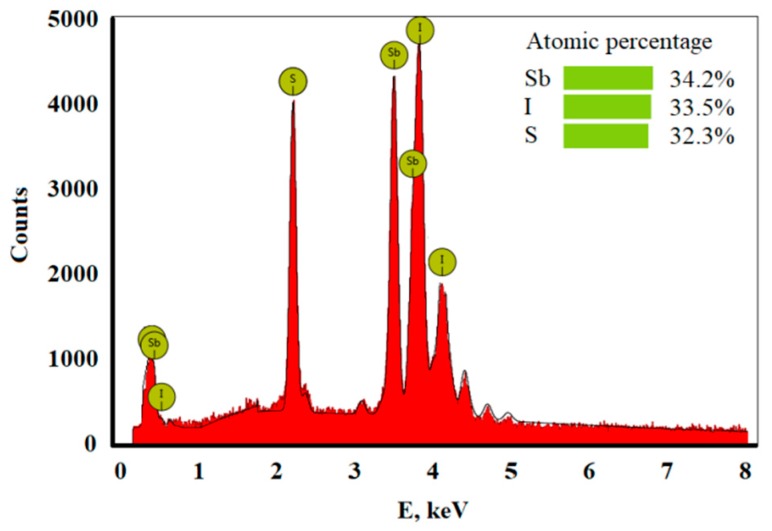
The energy-dispersive X-ray spectroscopy (EDS) spectrum of sonochemically prepared SbSI nanowires.

Figure 1c,g presents the typical scanning electron microscopy (SEM) micrographs of the sonochemically-prepared SbSI and the fabricated epoxy resin/SbSI nanowire composite plate, respectively. An EDS analysis of the SbSI nanowires was also performed. Characteristic peaks for antimony, sulphur, and iodine were observed (Figure 2). This confirms an elemental atomic ratio of 0.342:0.323:0.335 for Sb, S, and I, respectively, and indicated, within experimental error, that a stoichiometric antimony sulpho-iodide material was synthesized. The chemical route of SbSI nanowire growth and the details of the morphology, crystallographic structure, and chemical composition of the sonochemically produced SbSI xerogel have been previously presented, in [12,27].

**Figure 3:**
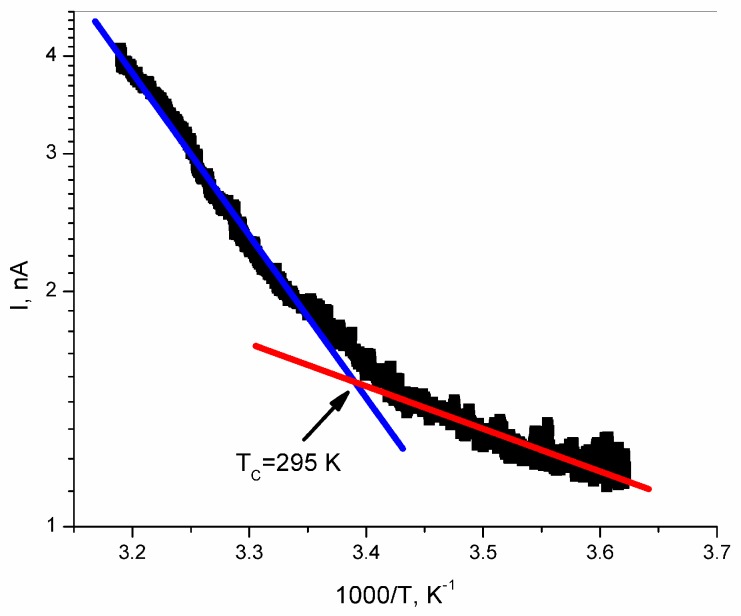
The temperature dependence of the electric current intensity for an epoxy resin/SbSI nanowire composite with an applied field of *E* = 1.7 × 10^5^ V/m. Red and blue solid curves represent the fitted relationship from Equation (1) in the ferroelectric and paraelectric phases, respectively; the arrow shows the temperature of the ferroelectric transition in the investigated composite. The values of the fitted parameters are noted in the following text.

Figure 3 shows the temperature (*T*) characteristics of the electric current intensity *I* in the epoxy resin/SbSI nanowire composite. The observed results (registered for cooling of the sample) are identical to the ones measured during heating. The obtained dependences were fitted using a least-squares approach in both rectilinear regions (which are identified with para- and ferroelectric phases in antimony sulpho-iodide) using the following equation:

(1) $$I=I_{0}\exp\left(-\frac{E_{A}}{k_{B}T}\right)$$

where *E_A_* is the activation energy of conductivity, *I*_0_ is the proportionality factor, and *k_B_* is the Boltzmann constant. The fitted values for *E_A_* for the cooling sample in the paraelectric and ferroelectric phases equal *E_Ap_* = 0.440 (12) eV and *E_Af_* = 0.084 (10) eV, respectively.

**Figure 4:**
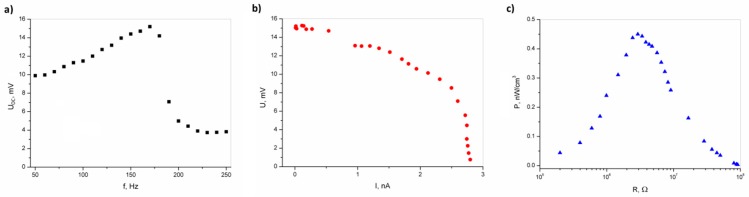
(**a**) Output voltage of an open circuit of epoxy resin/SbSI nanowire generator, registered for varying frequencies of sound excitation; (**b**) output voltage of epoxy resin/SbSI nanowire generator vs current intensity, registered for different load resistances (*f* = 170 Hz; *L*= 90 dB); (**c**) power density calculated for the volume of an epoxy resin/SbSI nanowire generator, as a function of load resistance with sound excitation (*f* = 170 Hz; *L* = 90 dB).

Figure 4a shows the output voltage of an open circuit for an epoxy resin/SbSI nanowire generator, registered for varying frequencies of sound excitation. The epoxy resin/SbSI nanowire composite voltage response increases with an increase in the frequency (*f*) of the acoustic signal, reaching a maximum of *f* = 170 Hz, and then decreases. Figure 4b presents the U-I characteristic of epoxy resin/SbSI nanowire generator registered for varying load resistances (*f* = 170 Hz; *L* = 90 dB). The shape is typical for an electromotive force source. The power density calculated for the volume of the epoxy resin/SbSI nanowire generator (Figure 4c) was determined by measuring the voltage, *U*, for various load resistances, *R* (corrected for the Princeton EG&G 5110 input impedance of 100 MΩ). The maximum power density output of the epoxy resin/SbSI nanowire generator equals 0.45 nW/cm^3^ for a load resistance (*R* = 2.9 MΩ) comparable with the source impedance. The power density values were calculated by dividing the absolute power by the sample dimensions. 

**Figure 5:**
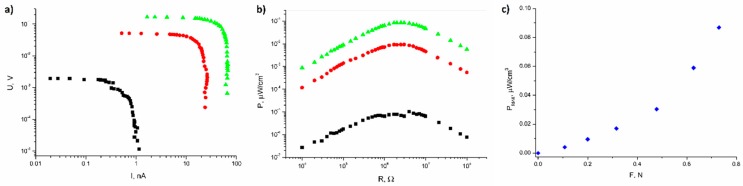
(**a**) The output voltage of epoxy resin/SbSI nanowire generator vs the current intensity for a vibration excitation (*f* = 24 Hz; *A* = 1 mm). (**b**) The power density calculated for a volume of epoxy resin/SbSI nanowire generator as a function of load resistance. The dependencies are presented for three chosen squeezing forces of a sample into a vibrating plate (■: *F* = 0 N; ●: *F* = 0.20 N; ▲: *F* = 0.73 N). (**c**) Maximum values for the power density registered for varying squeezing forces.

Figure 5 presents the results achieved for the epoxy resin/SbSI nanowire generator mounted on a vibrating plate. The measurements were performed over a narrow range of frequencies (16–24 Hz), which were limited by the vibrometer capabilities. All results presented in Figure 5 were measured for a frequency of 24 Hz, with the vibration amplitude *A* = 1 mm, and for various forces pressing a sample into a vibrating plate. Figure 5a shows the U-I characteristic of the epoxy resin/SbSI nanowire generator, obtained for varying squeezing forces (■: *F* = 0 N; ●: *F* = 0.20 N; ▲: *F* = 0.73 N). Their shapes are also typical for a source of electromotive force, as in the case of sound excitation. The power density calculated, for a given volume of epoxy resin/SbSI nanowire generator (Figure 5b), was also determined in the same way as in the case of sound excitation. The maximum power density output for the epoxy resin/SbSI nanowire generator was equal to 0.86 µW/cm^3^ (*F* = 0.73 N) for a load resistance (*R* = 2.5 MΩ) comparable with source impedance. One can see that an increase in the pressing force on the sample into the vibrating plate results in an increase in the power density (Figure 5c). 

**Figure 6:**
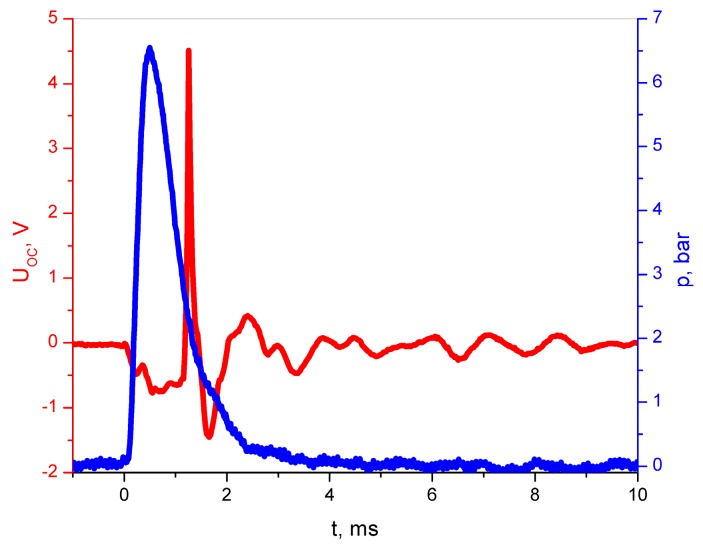
Open-circuit voltage of the epoxy resin/SbSI nanowire generator induced by shock pressure (red curve). The blue curve represents the pressure change of the CO_2_ shock wave.

Figure 6 presents the maximum magnitude, showing a typical transient characteristic of the open-circuit voltage, generated in an epoxy resin/SbSI nanowire active layer of the prototype generator. The voltage for the peak is higher than 4.5 V, in the case of the CO_2_ shock wave, with approximately 130 m/s velocity and a pressure of 6 bars. 

**Figure 7:**
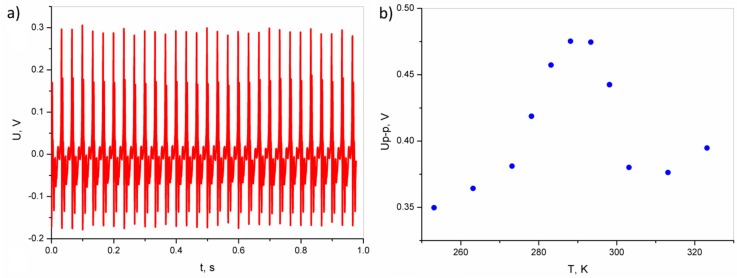
(**a**) Open circuit voltage signals registered by the epoxy resin/SbSI nanowire composite under impact (*f* = 30 Hz; *T* = 293 K; *F* = 17.8 N). (**b**) The open-circuit peak-to-peak voltage of epoxy resin/SbSI composite vs temperature (*f* = 30 Hz; *F* = 17.8 N).

Figure 7a presents the response of the composite to impact by a moving shaker tip (*f* = 30 Hz; *T* = 293 K; *F* = 17.8 N). One can see good recurrence of the signal. The discrepancy of the value for each peak is associated with various strains of the nanogenerator during the compressing and releasing stages. As shown in Figure 7b, the maximum generated voltage is reached at a temperature of ~293 K, which is comparable with the Curie temperature of the SbSI nanowires. For the temperature range above and below the Curie temperature, the generated voltage is about 20% lower than at the peak maximum.

## 4. Discussion

The sonochemically-produced SbSI can be classified (after drying) as an xerogel, composed of nanowires with average lateral dimensions of 10–100 nm, average lengths up to several micrometres (Figure 1c), and an elemental atomic ratio of antimony, sulphur and iodine (0.342:0.323:0.335), indicating the presence of stoichiometric antimony sulpho-iodide (Figure 2). For the case of the obtained epoxy resin/SbSI nanowire composite, one can see a uniform distribution of SbSI nanowires throughout the whole sample volume (Figure 1g). This proves that a well homogenized mixture is obtained when mixed by ultrasound irradiation. Considering the nanocomposite classification presented in [28], an epoxy resin/SbSI nanowire composite can be categorized as a so-called 0–3-type composite, in which nanoparticles are randomly dispersed within a matrix. Epoxy resin forms a matrix, while dispersed SbSI nanowires readily touch each other, even at low volume fractions. Therefore, continuous percolation of nanowires throughout the sample can be achieved. 

The SbSI nanowires/epoxy resin composite was prepared with differing mass ratios. With an increasing number of SbSI nanowires in the composite, the registered signal increases up to a mass ratio of 1:4. A further increase in the number of SbSI nanowires results in conglomeration of the nanowires. For SbSI nanowire content over 50%, the mechanical properties of composite get worse. A similar observation has been noted for an FRP (Fiber Reinforced Polymer) laminate with integrating strain sensor containing SbSI nanowires [7]. Details of these studies and a comparison of the results are being prepared for a separate future manuscript.

Analysing the electric conductivity of epoxy resin/SbSI nanowire composite (Figure 3), one can see two straight ranges with different slopes. The change of the slope of *I* (1/*T*) is related to the phase transition in the SbSI nanocrystals (dispersed in the epoxy resin), between the para- and ferroelectric regions. The value of the activation energy of the epoxy resin/SbSI nanowire composite was obtained for the first time, and can only be compared with values for the SbSI gel [29]. Results presented in this paper (*E_Ap_* = 0.440 (12) eV and *E_Af_* = 0.084 (10) eV in the paraelectric and ferroelectric phases, respectively) differ when compared to the SbSI gel (*E_Ap_* =0.3342 (1) eV and *E_Af_* = 0.2623 (4) eV in the paraelectric and ferroelectric phases, respectively) [29]. The difference between activation energies in the para- and ferroelectric phases reported in this paper is much higher than for the pure SbSI nanowires. This is caused by a significant difference in the values of the activation energy in the ferroelectric phases of both materials, indicating a stronger interaction between the SbSI nanowires and the epoxy resin matrix in the ferroelectric phase when compared to the paraelectric phase. This can also be associated to the complicated structure of the electron levels of SbSI and the presented composite. This thesis can be verified in future work by using deep-level transient spectroscopy (DLTS) and X-ray photoelectron spectroscopy (XPS).

Figure 4, Figure 5, Figure 6 and Figure 7 present the electric responses of the composite to different types of excitation: sound, vibration, shock wave, compression, and impact. These types of excitation can practically occur in the potential applications of this material, and take place in everyday environments. The output voltage for the open circuit of an epoxy resin/SbSI nanowire generator, registered for different frequencies of sound excitation, reaches its maximum at *f* = 170 Hz. This value represents the resonance frequency, which is dependent on the stiffness coefficient and the mass of the sample. The first factor is influenced by the flexibility and relaxation delay of the epoxy resin, which is mainly responsible for the elastic properties of the composite. This is a common phenomenon for nanogenerators [16]. The maximum power density and output voltage obtained for sound excitation are 0.45 nW/cm^3^ and 0.0153 V, respectively. The same properties were determined for excitation by vibration. However, the obtained values were much higher in this case. Vibrations are important sources of energy, which can be harvested for driving small electronic components. However, most ambient vibrations have a wide frequency spectrum range. Moreover, this range may even drift over time. Therefore, an effective nanogenerator to harvest this type of energy needs to meet at least two criteria [30]. First, it can be applicable to a broad vibration frequency range of approximately 1–300 Hz, instead of being fixed to a single resonance frequency. Second, it can effectively respond to vibrations at a low frequency (below a few hundred Hz), due to the fact that most of ambient vibrations fit within this range. Human motions generate natural oscillation frequencies below a few dozen Hz, while vibrations induced by machines lie in the range up to few hundred Hz [31]. For the presented nanogenerator, the maximum power density and output voltage obtained for vibrations are 860 nW/cm^3^ and 0.166 V, respectively. This is achieved at a frequency of 24 Hz, which is close to the vibration frequency generated by both humans and machines. One can see in Figure 5b,c that an additional pressure force leads to an increase in the maximum power density, due to improved mechanical contact between the vibrating plate and the sample, giving a more efficient conversion of mechanical vibration energy to electric energy. For real-world applications, the nanogenerator can be mounted between structural elements of a given machine, which would improve the mechanical contact and simultaneously increase the value of output power.

In Table 1, one can see the comparison of voltages and power densities registered for different composites (consisting of SbSI, BaTiO_3_, and ZnO) and PZT (nanofibers and textile). The obtained results for the sound excitation are smaller for epoxy resin/SbSI nanowire composites than for a cellulose/SbSI composite. Conversely, the power density is significantly higher in the case of excitation by vibrations. This leads to promising applications for harvesting this type of energy. One can see that for shockwave depressurization, the voltage generated by the epoxy resin/SbSI nanowire composite is slightly over 8 times lower than for pure SbSI nanowires; however, this is achieved at nearly 10 times lower pressure. Table 1 shows that the parameters of the nanogenerators made from epoxy resin/SbSI nanowire composites are comparable with those of other devices. However, the presented parameters (voltage, power) are difficult to compare to different piezoelectric nanogenerators, due to the varying excitation methods (i.e., bending, pressing, knocking, and ultrasonic waves); lack of information about parameters of the used excitation; and different power calculation methods. 

Taking into consideration the Curie temperature, estimated for the composite to be *T* = 295 K (Figure 3), one can expect the collapse of the piezoelectric properties at higher temperatures. However, as shown in Figure 7b, an increase in the temperature over the Curie point does not cause a collapse in the signal. In the temperature range above and below the Curie temperature, the generated voltage is only about 20% lower than at the peak. This can be explained by the coexistence of both ferro- and paraphases in the SbSI crystals. This phenomenon is known for ferroelectric semiconductors, and has been shown in [37]. The temperature range for the coexistence of both phases depends on the carrier concentration [38]. For the presented composite, this range is wide, and even for temperatures 20 K higher than Currie point, the signal is not significantly smaller (Figure 7b). Moreover, other results may indicate the coexistence of para- and ferrophases in SbSI, as given in [39]; non-zero remnant polarization in the SbSI layer was found up to 313 K. Furthermore, the Landau theory and Kern formulation predict shifting of the Curie temperature to higher temperatures under applied strain. Experimental results for the Curie temperature shift with strain in low-dimensional SbSI, as presented in [40]. The Curie temperature was increased by 60 K from around 298 K for the unstrained crystal, by applying a mechanical strain [40]. Broadening of the working temperature range of the presented composite may be achieved by doping the SbSI. Substitution of 0.1% sulphur elements with chlorine shifts the Curie temperature to 330 K [41]. Similarly, partial substitution of sulphur elements with oxygen shifts the Curie point to 338 K [42]

## 5. Conclusions

For the first time, an epoxy resin composite incorporating antimony sulpho-iodide (SbSI) nanowires is presented. The preliminary investigations of the epoxy resin/SbSI nanowire composite showed that the fabricated composite is a promising and suitable material for construction of nanogenerators and nanosensors.

A simple device containing one pair of electrodes in a sandwich configuration allows the generation of an open-circuit voltage of 0.0153 V_RMS_, 0.166 V_RMS_, 4.51 V_p-p_ and 0.475 V_p-p_ under sound excitation (*f* = 175 Hz; *L* = 90 dB), vibrations (*f* = 24 Hz; *A* = 1 mm; *F* = 0.73 N), and shock pressure (*p* = 6 bar), and impact (*f* = 30 Hz, *T* = 293 K, *F* = 17.8 N), respectively. The maximum output power densities of 0.45 nW/cm^3^ and 860 nW/cm^3^ have been achieved for excitation using sound and vibration, respectively. 

It must be noted that the functional parameters (voltage, current, and power) presented in this article are difficult to compare to data reported for other piezoelectric nanogenerators. Different researchers have applied various excitation methods (i.e., bending, pressing, and ultrasonic waves). Additionally, very often the power of the utilized excitation method is not presented. However, one can recognize (Table 1) that the parameters of the nanogenerators made from epoxy resin/SbSI nanowire composites are comparable with those of other devices.

Further improvements of the functional parameters could be achieved by optimizing the size of the samples (thickness and electrodes area) and the device’s connections. Moreover, various materials for electrodes, as well as their different geometries (sandwich, planar, interfringe), should be considered to improve the functional parameters of the epoxy resin/SbSI nanowire composite. Revised devices can also be created, i.e., for the detection of vibration, harvesting different forms of mechanical energy.

The application of ultrasound irradiation makes the presented material easy, low-cost, and effective in production. Similar composites of SbSI nanowires with other epoxy resins (with different viscosity) as well as with polymers (i.e., PMMA, PVP) will be fabricated, and their corresponding manuscripts are planned to be published. The application of epoxy resin with lower viscosity and the use of an external electric field during cross-linking should allow ordering of the *c*-axis of SbSI nanowires in the direction of the field. The SbSI nanowires can be properly oriented to form nanocomposites with anisotropic, piezoelectric properties. 

## Figures and Tables

**Table 1:** Comparison of open-circuit voltages and power densities registered for different types of materials, consisting of SbSI, BaTiO_3_, ZnO, and PZT.

Material	U_OC_	Max *P*	Excitation	References
cellulose/SbSI composite	0.024 V_RMS_	41.5 nW/cm^3^	sound (*f* = 175 Hz, *L* = 90 dB)	[9]
	2.5 V_p-p_	-	shock wave (*p* = 30 bar)	
PAN/SbSI composite	0.4 V_p-p_	-	bending (*f* = 1 Hz)	[32]
SbSI nanowires	29.0(7) V_p-p_	-	shock wave (*p* = 59 bar)	[10]
epoxy resin/SbSI nanowires composite	0.166 V_RMS_	860 nW/cm^3^	vibrations (*f* = 24 Hz; *A* = 1 mm; *F* = 0.73 N)	this article
	0.0153 V_RMS_	0.45 nW/cm^3^	sound (*f* = 170 Hz, *L* = 90 dB)	
	4.51 V_p-p_	-	shock wave (*p* = 6 bar)	
	0.475 V_p-p_	-	impact (*f* = 30 Hz, *T* = 293 K, F = 17.8 N)	
SbSI/PMMA	4.7 V_p-p_	550 nW/cm^2^	pressing (*F* = 2 N)	[33]
BaTiO_3_/bacterial cellulose	23 V_p-p_	640 nW/cm^2^	compressive stress	[34]
BaTiO_3_/PDMS	3 V_p-p_	40 nW/cm^2^		
BaTiO_3_/bacterial cellulose	1.9 V_p-p_	-	bending (*f* = 1 Hz)	
ZnO NW/PVDF	0.4 V_p-p_	-	pressing (*f* = 1 Hz)	[35]
	0.06 V_p-p_	-	bending (*f* = 0.2 Hz)	
PZT nanofiber	0.6 V_p-p_	-	periodic knocking	[17]
	0.6 V_p-p_	-	pressing	
PZT textile	0.4 V_p-p_	-	bending	[36]

RMS subscript refers to root mean square amplitude, and p-p subscript is the peak-to-peak amplitude.
